# Allantoin Derived From Dioscorea opposita Thunb Ameliorates Cyclophosphamide-Induced Premature Ovarian Failure in Female Rats by Attenuating Apoptosis, Autophagy and Pyroptosis

**DOI:** 10.7759/cureus.50351

**Published:** 2023-12-11

**Authors:** Xiaolan Wang, Peipei Yuan, Mengnan Zeng, Mo Sun, Xiaoyang Wang, Xiaoke Zheng, Weisheng Feng

**Affiliations:** 1 Academy of Chinese Medical Sciences, Henan University of Chinese Medicine, Zhengzhou, CHN; 2 School of Pharmacy, Henan University of Chinese Medicine, Zhengzhou, CHN

**Keywords:** pyroptosis, autophagy, apoptosis, granulosa cells, premature ovarian failure, allantoin

## Abstract

Background and objectives

Cyclophosphamide (CP) is widely used as a chemotherapy drug for the treatment of malignant tumors and autoimmune diseases, but it has strong toxic and side effects and can cause permanent damage to the ovaries, which affects women's quality of life. This study aimed to investigate the anti-premature ovarian failure protective effect of allantoin isolated from *Dioscorea opposita* Thunb.

Methods

Firstly, 75 mg/kg CP was injected into rats to establish an* in vivo* model of premature ovarian failure (POF). The POF rats were divided into the normal control group (NC), premature ovarian failure group (POF), and POF group treated with allantoin (ALL I 140 mg/kg and ALL II 70 mg/kg, daily 21 days). It investigated the estrous cycles, hormone levels, apoptosis rate, mitochondrial membrane potential (MMP), reactive oxygen species (ROS), mitophagy, and protein marker (Bax, Bcl2, LC3B, L-1β, caspase-1 and NLRP3).

Results

The results indicated that allantoin alleviated cyclophosphamide-induced premature ovarian failure in female rats, decreased the anoestrum, increased the level of estradiol (E2), and decreased the levels of follicle-stimulating hormone (FSH) and luteinizing hormone (LH), decreased apoptosis rate, MMP, mitophagy and ROS in ovarian granulosa cells of POF rats, down-regulated L-1β, caspase-1, LC3B-II/LC3B-I in ovarian tissue, and up-regulated the Bcl2 and NLRP3.

Conclusions

Our study revealed the ovarian-protective effect of allantoin in CP-induced premature ovarian failure for the first time, the effect was achieved through attenuation of the apoptosis, autophagy, and pyroptosis. The study underlines the potential clinical application of allantoin as a protectant agent for premature ovarian failure.

## Introduction

Premature ovarian failure (POF) is a prevalent disorder in the gynecological endocrine system. In recent years, there has been an increase in POF incidence among reproductive-age women, and it has been linked to chemotherapy exposure in systemic lupus erythematosus and cancer patients [1,2]. Cyclophosphamide (CP) can disrupt ovarian function by inhibiting hormone secretion [3] and increasing oxidative stress, apoptosis, and inflammation [4,5]. Therefore, Further studies are necessary to comprehend the molecular mechanism and alterations in gene expression under chronic impaired conditions following CP treatment, which may contribute to preventing the prolonged toxic effects. Exposure to CP directly and indirectly triggers apoptosis by inducing DNA damage, inhibiting proliferation, and causing mitochondrial dysfunction, ultimately leading to a reduction in ovarian reserve [6]. Granulosa cells (GCs) are crucial for maintaining follicle development and also secrete reproductive hormones, which are closely related to premature ovarian failure. GCs are the target cells of CP and are widely used in evaluating the reproductive toxicity of drugs [7]. The occurrence of diseases is often the result of the interaction of multiple cell death modes [8]. Apoptosis and autophagy of GCs play an important regulatory role in the process of ovarian follicular atresia in animals, and recent studies have shown that pyroptosis is also involved in the process of ovarian follicular atresia [9]. Apoptosis, autophagy, and pyroptosis achieved a subtle balance in the actual treatment, which needs further research [10].

Allantoin (ALL) is the main active constituent of the *Dioscorea opposita* Thunb [11], which has several effects, including anti-irritating [12], anti-inflammatory [13], promoting cell growth [14], and so on. Recently, our team found that allantoin has an estrogen-like effect through the expression of ERα and GPR30 in uterine tissue [15]. Researchers have found that natural phytoestrogens have a positive effect on ovarian follicular development in rats [16,17]. Therefore, in this study, allantoin was used to treat a cyclophosphamide-induced premature ovarian failure rat model, and the mechanism of its effects on POF was discussed.

## Materials and methods

Analysis of allantoin

Allantoin was isolated from the *Dioscorea opposita* Thunb, with 20% methyl alcohol as the mobile phase. The allantoin sample was analyzed through the chromatographic column (Phenomenex Synergi Hydro-RP, 4μm, 80A, 250×4.6 mm) of the HPLC (Waters e2695 separations Module), determine wavelength: 254 nm, mobile phase: methyl alcohol: H2O (5:95), flow velocity:1 mL/min, column temperature: 30℃, sample size: 10 μL, and the retention time was recorded.

Chemicals

Cyclophosphamide (CP) was purchased from Sdsensor Pharmaceutical Co., Ltd. (Jiangsu, China). Oestradiol valerate was purchased from Xianling Pharmaceutical Co., Ltd (Guangzhou, China). All other chemicals were analytical grade.

Animals and experimental design

Fifty Sprague-Dawley (SD) rats (female, 180-220 g) were purchased from Charles River Laboratory Animal Technology Co., Ltd. (Beijing, China) (certificate no. SCXK (Jing)-2016-011). The rats were maintained under standard laboratory conditions (temperature: 25 ± 2°C, humidity: 60 ± 5%, 12-h dark/light cycle). All the rats were given a one-week acclimatization period before the experiment and had adequate water and feed all the time. All the animal experiments were approved by the Committee of Animal Use and Protection of Henan University of Chinese Medicine (DWLL2018080003).

The rats were randomly assigned into five groups: the normal control (NC) group, premature ovarian failure (POF) group, estradiol valerate (EV) group, allantoin I (ALL I, 140 mg/kg) group, allantoin II (ALL II, 70 mg/kg) group (each group n = 10). All rats were treated once per day for 21 consecutive days. The POF group rats were induced by the intraperitoneal injection of 75 mg/kg CP [18]. Allantoin was dissolved in distilled water, and the NC and POF groups were treated with the same volume of distilled water as the treated groups. In the EV group, the POF rats received EV at a dose of 0.08 mg/kg. In the ALL I and ALL II groups, the POF rats respectively received allantoin at a dose of 140 mg/kg and 70 mg/kg. The vaginal smear samples of the rats were taken at a fixed time every day. After the last treatment, the rats fasted for 12 hours. The rats were anesthetized with sodium pentobarbital (35 mg/kg), and blood plasma samples were collected by abdominal aortic method, rat ovaries were collected to determine the next detection, and the ovarian granulosa cells of rats were isolated by trypsin method [19].

Measuring the estrous cycles of the rats

Cytological examination of vaginal exfoliation [20] was used to measure the estrous cycles. Normal saline (60 μL) was drawn using a pipette and was gently inserted 3-5 mm into the vagina of rats; then, it was blown two to five times until the liquid changed to cloudy. Then drops of the collected liquid were placed on a slide, the liquid was dried fully and stained with hematoxylin-eosin (HE) stain.

Hormonal assays

After centrifugation at 3500 rpm for 10 min, the serum of rats was collected. E2, FSH, and LH were quantitatively estimated using enzyme-linked immunosorbent assay (ELISA) kits (Bioswamp Biological Technology Co., Ltd, Wuhan, China).

Morphological evaluation

The ovaries were stored in 10% formalin, and then the tissues were embedded in paraffin, sliced at a thickness of 5 µm, and stained with HE. Pathological changes in the ovaries were assessed and photographed under a microscope (Olympus BX-51, Japan).

Annexin V-7AAD (7-amino-actinomycin)/Annexin V-PE double staining assay

The isolated granulosa cells of each group were washed with phosphate-buffered saline (PBS) and apoptosis was detected by Annexin V-PE Apoptosis Detection Kit (BD Pharmingen^TM^, USA). The single-cell suspension stained with 5 µL Annexin V-7AAD (7-amino-actinomycin) and 5 µL Annexin V-PE working solution, mixed well. After that, the cells were allowed to stand for 15 min at room temperature in the dark and detected by flow cytometry (BD, FACSAria III, USA) within 1 h. Green fluorescence detection of Annexin V-7AAD was performed via the fluorescein isothiocyanate (FITC) channel (7-AAD), and red fluorescence detection of propidium iodide was performed via the PI channels (PE). The results were analyzed by FlowJo 10.8.1 software (BD Biosciences, USA).

Mitochondrial membrane potential (MMP) staining assay

The MMP was stained with JC-1 (Beyotime, China) and detected by flow cytometer via the FITC channel and PE channel. The cells delineated in region Q2/Q3 were the levels of MMP, the results were analyzed by FlowJo 10.8.1 software.

Reactive oxygen species (ROS) staining assay

ROS level was measured by using dichlorodihydrofluorescein diacetate (DCFH-DA) reactive oxygen ROS fluorescent probe (Solarbio, China), isolated granulosa cells were incubated with DCFH-DA at a final concentration of 10 µM in the darkness for 30 min at 37℃. After the cells were washed three times with PBS. ROS production was evaluated by the flow cytometer. The level of intracellular ROS was shown as a percentage of non-treated control, the results were analyzed by FlowJo 10.8.1 software.

Mitophagy staining assay

The isolated granulosa cells of each group were co-stained with Mitophagy Red™ and MitoLite™ Green (AAT Bioques, USA) working solutions at 37℃ for 30 min. The cells were collected and washed with PBS and then resuspended in PBS. The cell fluorescence intensity was detected by Flow Sight cytometry (Amnis, Germany). The cells were imaged using a fluorescence microscope with a Cy3/TRITC filter for Mitophagy Red™ and FITC filter for MitoLite™ Green FM and overlapped with each other, the results were analyzed by IDEAS 6.2 software (Amnis, Germany).

Western blotting

Protein was extracted from tissues following the instructions of the commercial kit (Beyotime, China), and the concentration was determined by using a BCA protein assay kit (Solarbio, China). Equal amounts of protein samples were separated by sodium dodecyl sulfate-polyacrylamide gel electrophoresis (SDS-PAGE and transferred to a polyvinylidene fluoride (PVDF) membrane. The membrane was blocked in 5% (v/v) non-fat milk in tris-buffered saline (TBST) for 2 h at room temperature and then blotted with primary antibody, Bax and Bcl2(1:1000; Invitrogen, USA), LC3B (1:1000, Cellsignal, USA), IL-1β, caspase-1 and NLRP3 (1:1000; Abcam, UK), β-actin (1:3000, Proteintech, China) overnight at 4°C. After washing in TBST, the membrane was incubated with a secondary antibody for 1 h at room temperature. The intensity of the proteins was quantified using Odyssey near infrared fluorescence quantification system (Li-cor, USA).

Transmission electron microscopy (TEM)

In order to analyze autophagic vacuoles in the ovary cells, ovaries were sectioned in blocks of 1x1 mm thick a scalpel blade, fixed in cold 0.25% glutaraldehyde in 0.1 M phosphate buffer (pH 7.0-7.5) during 2-4 h, and then transferred to fresh phosphate buffer three times. Sections were subsequently post-fixed in 2% osmium tetroxide containing 0.1 M phosphate buffer (pH 7.4) for 2 h, 0.1 M phosphate buffer washing three times, dehydrated in graded alcohols and propylene oxide and embedded in araldite for 12 h (37℃), sliced up into 60-80 nm ultrathin section using an ultramicrotome. The cuts were mounted on copper grids and contrasted with uranyl acetate (2%) and lead citrate, dried at room temperature. The cuts were observed in transmission HT7700 electron microscope (Hitachi, Japan).

Statistical analysis

The results are presented as the mean ± SD, and statistical analyses were conducted using GraphPad Prism 8.0 (GraphPad Software, San Diego, CA, USA), and comparisons between groups were performed by one-way ANOVA, whereas multiple comparisons were performed using the LSD method, p < 0.05 were considered statistically significant.

## Results

Structure and HPLC analysis of allantoin

**Figure 1:**
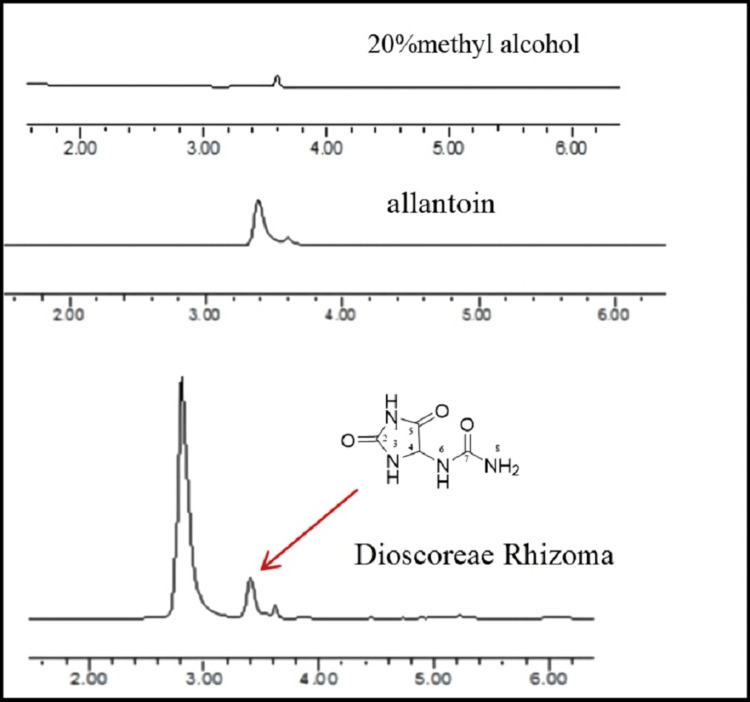
Liquid chromatogram and structural formula of allantoin

The structural formula and liquid chromatogram of allantoin are shown in Figure 1. A single peak at 3.4 min was obtained representing allantoin and the peak at 3.6 min was of 20% methanol solvent peak.

Effects of ALL on estrous cycles 

**Figure 2:**
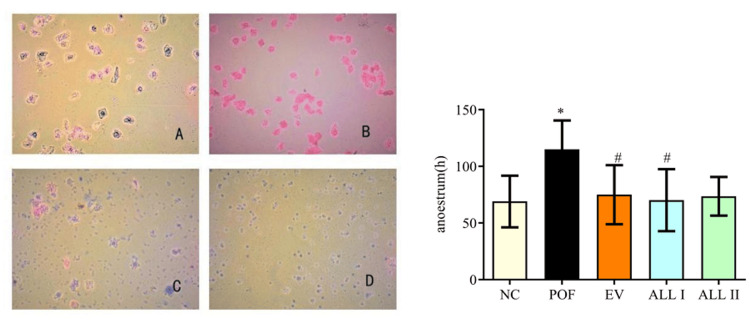
Effects of ALL on anoestrum of POF rats Data are presented as means ± SD (n = 10). ^*^p < 0.05, ^**^p < 0.01 compared with the NC group; ^#^*p* < 0.05, ^##^*p* < 0.01 compared with the POF group. A :proestrum; B:oestrum; C: metestrus; D :anoestrum NC: normal control group; POF: premature ovarian failure group; EV: estradiol valerate group; ALL I: the 140 mg/kg dose of allantoin group; ALL II:  the 70 mg/kg dose of allantoin group.

We have determined the treatment of CP can cause ovarian injury and premature ovarian failure in rats. The ovarian function was evaluated by the observations on the estrous cycle, endocrine function, follicle development, and ovary histopathological changes. As shown in Figure 2, the examination of estrous cycles was detected by hematoxylin and eosin (HE) staining of vaginal smear. We observed the anoestrum was lengthened in the POF group compared with the NC group (p < 0.05). ALL I group could shorten the anoestrum (p < 0.05).

Effects of ALL on hormone levels in the serum of POF rats

**Figure 3:**
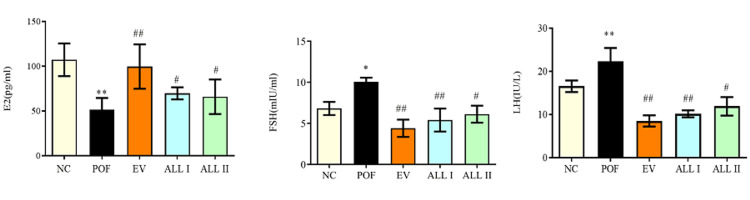
Effects of ALL on hormone levels in the serum of POF rats Results are presented as means ± SD (n = 10).^*^p < 0.05, ^**^p < 0.01 compared with the NC group; ^#^*p* < 0.05, ^##^*p* < 0.01 compared with the POF group. NC: normal control group; POF: premature ovarian failure group; EV: estradiol valerate group; ALL I: the 140 mg/kg dose of allantoin group; ALL II:  the 70 mg/kg dose of allantoin group.

Figure 3 shows the levels of hormones in the serum of each group. The levels of E2 were significantly reduced in the POF rats compared with the NC group (p < 0.01). FSH and LH levels were increased compared with those in the NC group (p < 0.05). Compared with the POF group, ALL I, ALL II, and EV significantly increased the level of E2 and decreased the levels of FSH and LH (p < 0.01 or p < 0.05).

Effect of ALL on uterine histopathology

**Figure 4:**
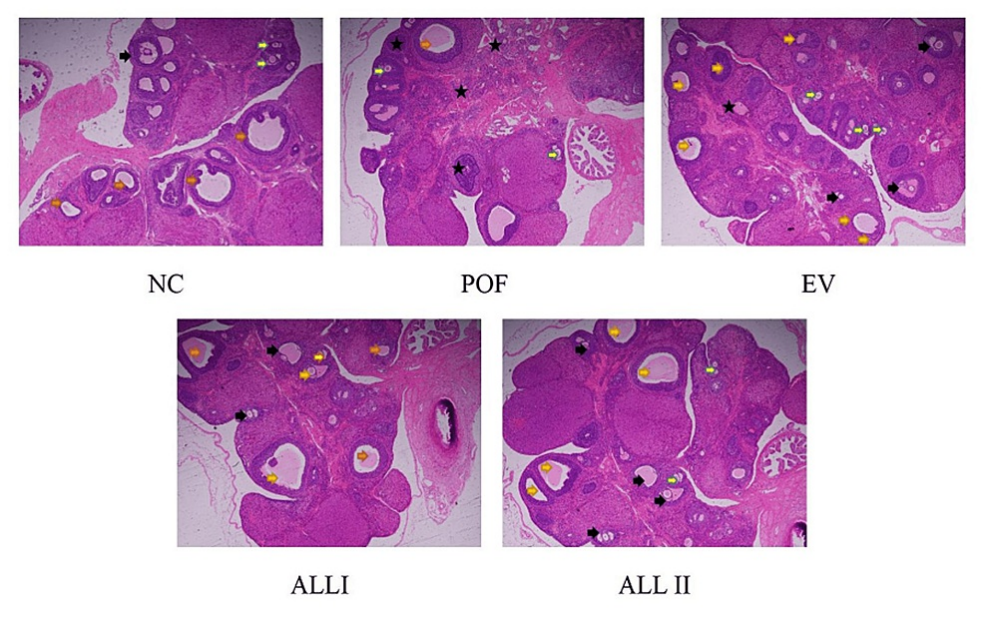
The effect of ALL on ovarian failure in rats The tissues were surgically excised and subjected to histological study by staining with hematoxylin & eosin (HE) stain (magnification 200X). NC: normal control group; POF: premature ovarian failure group; EV: estradiol valerate group; ALL I: the 140 mg/kg dose of allantoin group; ALL II:  the 70 mg/kg dose of allantoin group.

As shown in Figure 4, we observed the number of atretic follicles was significantly increased in the POF group compared with the NC group and there were few mature follicles, primordial follicles, and secondary follicles. After treatment with EV and ALL, there was an obvious reduction in the number of atretic follicles and an increase in the numbers of mature follicles, primordial follicles, and secondary follicles.

Effect of ALL on apoptosis rate in ovarian granulosa cells of POF rats 

**Figure 5:**
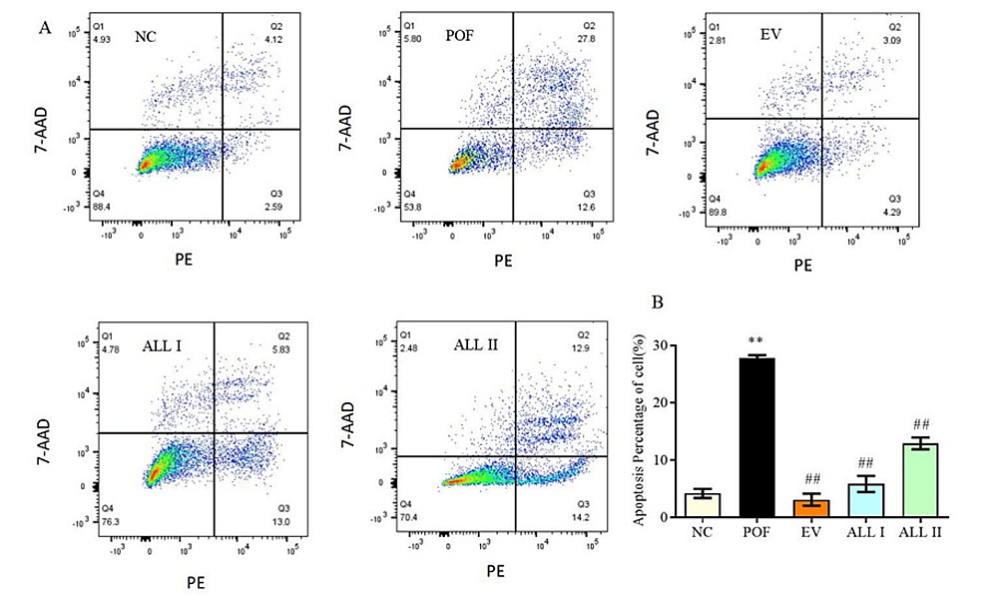
Effect of ALL on apoptosis rate in ovarian granulosa cells of POF rats Results are presented as means ± SD (n = 4).^*^p < 0.05, ^**^p < 0.01 compared with the NC group; ^#^*p* < 0.05, ^##^*p* < 0.01 compared with the POF group NC: normal control group; POF: premature ovarian failure group; EV: estradiol valerate group; ALL I: the 140mg/kg dose of allantoin group; ALL II:  the 70mg/kg dose of allantoin group

As shown in Figure 5, compared to the NC group, the percentage of apoptosis cells was obviously increased to 27.8% (p < 0.01) after being treated with PC. Compared to the POF group, the percentage of apoptosis cells in the treated groups obviously decreased to 3.09%, 5.89%, and 12.9% (p < 0.01).

Effect of ALL on mitochondrial membrane potential (MMP )in ovarian granulosa cells of POF rats

**Figure 6:**
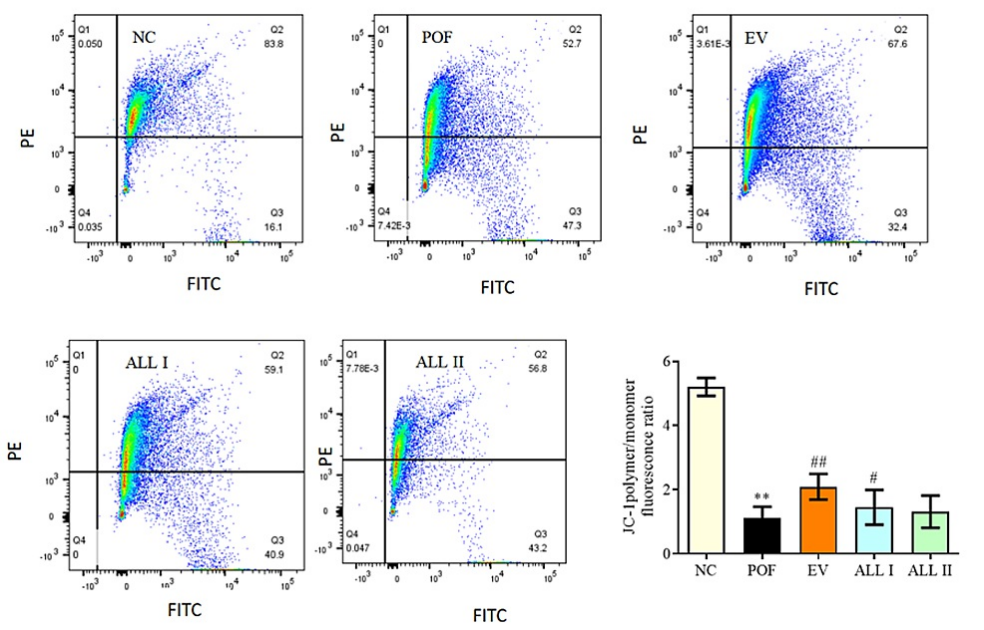
Effect of ALL on MMP of rat ovarian granulosa cells Results are presented as means ± SD (n = 4).^*^p < 0.05, ^**^p < 0.01 compared with the NC group; ^#^*p* < 0.05, ^##^*p* < 0.01 compared with the POF group. NC: normal control group; POF: premature ovarian failure group; EV: estradiol valerate group; ALL I: the 140 mg/kg dose of allantoin group; ALL II:  the 70 mg/kg dose of allantoin group; MMP: mitochondrial membrane potential.

As shown in Figure 6, the levels of MMP were evaluated by the JC-1 fluorescence ratio. Compared to the NC group, the levels of MMP were obviously decreased (p < 0.01) after treated with CP, and compared to the POF group, MMP were increased significantly (p < 0.05 or p < 0.01) after treated with the EV and ALL.

Effect of ALL on reactive oxygen species (ROS) level in ovarian granulosa cells of POF rats

**Figure 7:**
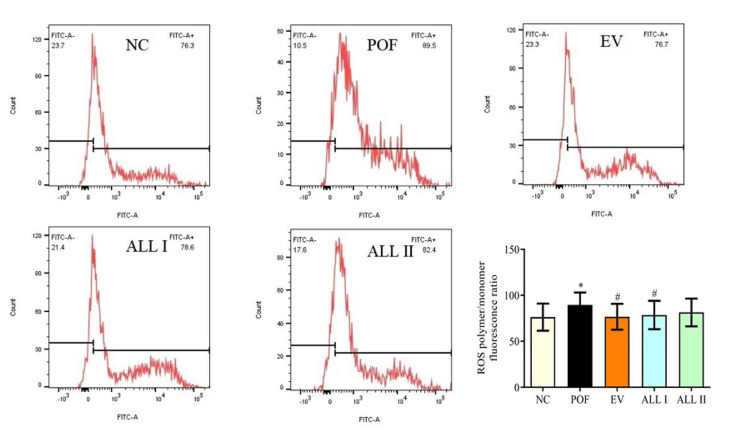
Effect of ALL on ROS in ovarian granulosa cells of POF rats Results are presented as means ± SD (n = 4).^*^p < 0.05, ^**^p < 0.01 compared with the NC group; ^#^*p* < 0.05, ^##^*p *< 0.01 compared with the POF group. NC: normal control group; POF: premature ovarian failure group; EV: estradiol valerate group; ALL I: the 140 mg/kg dose of allantoin group; ALL II:  the 70 mg/kg dose of allantoin group; ROS: reactive oxygen species.

As shown in Figure 7, after being exposed to 75 mg/kg CP, the level of ROS obviously increased to 89.5% after treated with CP (p < 0.05), and ROS was decreased to 76.7%, 78.6%, 82.4% after treated with EV and ALL, the EV and ALL I had a significant change compared with the POF group (p < 0.05).

Effect of ALL on mitophagy in ovarian granulosa cells of POF rats

**Figure 8:**
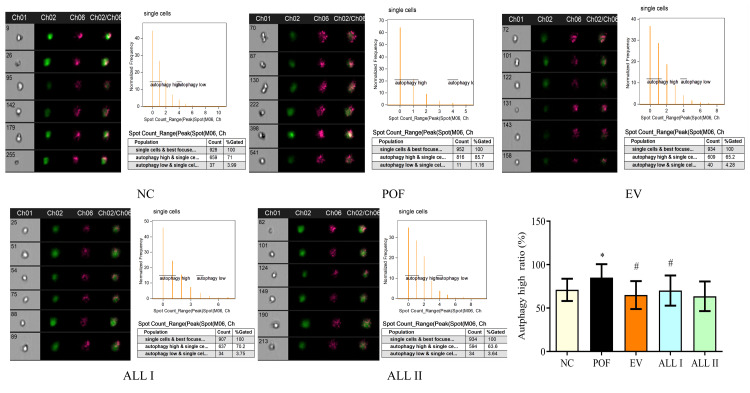
Effect of ALL on mitophagy in ovarian granulosa cells of POF rats Results are presented as means ± SD (n = 3).^*^p < 0.05, ^**^p < 0.01 compared with the NC group;^ #^*p* < 0.05, ^##^*p *< 0.01 compared with the POF group. CH01: Cell; CH02: Mitochondria stain; CH06: Autophagy stain; CH02/CH06: Merge NC: normal control group; POF: premature ovarian failure group; EV: estradiol valerate group; ALL I: the 140 mg/kg dose of allantoin group; ALL II:  the 70 mg/kg dose of allantoin group.

As shown in Figure 8, the mitochondria were labeled by green fluorescence, and autophagy was labeled by red fluorescence. A high ratio of autophagy was set as the level of mitophagy. Figure 8 shows cell real-time fluorescence staining of each group. The morphology and staining of cells can be observed distinctly. A high ratio of autophagy in each group was quantified. Compared with the NC group, mitophagy increased markedly after being treated with CP (p < 0.01). Mitophagy of EV and ALL I groups was decreased significantly compared to the POF group (p < 0.05).

Effect of ALL on protein expression of Bax, Bcl2, and LC3B-II/LC3B-I in the ovarian tissues of POF rats

**Figure 9:**
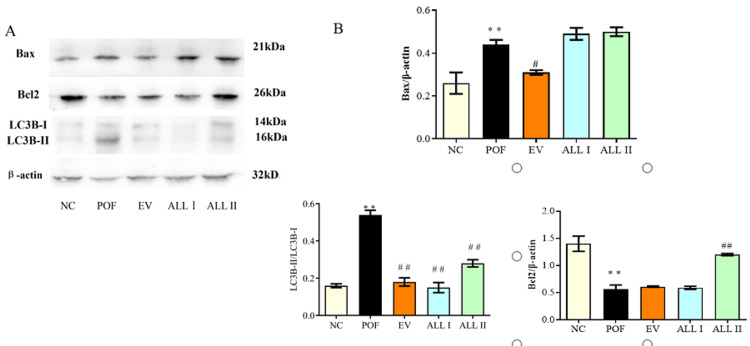
Effect of ALL on protein expression of Bax, Bcl2 and LC3BII/I in the ovarian tissues of POF rats Results are presented as means ± SD (n = 3).^*^p < 0.05, ^**^p < 0.01 compared with the NC group; ^#^*p *< 0.05, ^##^*p* < 0.01 compared with the POF group. NC: normal control group; POF: premature ovarian failure group; EV: estradiol valerate group; ALL I: the 140 mg/kg dose of allantoin group; ALL II:  the 70 mg/kg dose of allantoin group.

As shown in Figure 9, the results revealed that CP obviously increased the expression of Bcl2 and LC3B-II/LC3B-I and decreased the expression of Bax (p < 0.01). After treating the EV and ALL, the expression of LC3B-II/LC3B-I was reduced, but the ALL I groups' expression of Bax was not obviously changed (p >0.05 ), and the ALL II group's expression of Bcl2 was increased (p < 0.01).

Effect of ALL on the protein expression of IL-1β, caspase-1, and NLRP3 in the ovarian tissues of POF rats

**Figure 10:**
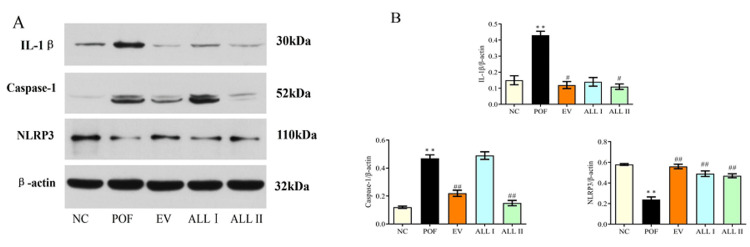
FIGURE 10: Effect of ALL on protein expression of IL-1β, caspase-1 and NLRP3 in the ovarian tissues of POF rats Results are presented as means ± SD (n = 3).^*^p < 0.05, ^**^p < 0.01 compared with the NC group; ^#^*p* < 0.05, ^##^*p* < 0.01 compared with the POF group. NC: normal control group; POF: premature ovarian failure group; EV: estradiol valerate group; ALL I: the 140 mg/kg dose of allantoin group; ALL II:  the 70 mg/kg dose of allantoin group.

As shown in Figure 10, there was a significant increase in the expression of L-1β and caspase-1 (p < 0.01) in the ovarian tissues of POF rats, and the results are out of the ordinary; there was a significant decrease in the expression of NLRP3 (p < 0.01) compared with the NC group. On the other hand, there was a significant decrease in the expression of L-1β (p < 0.05) and caspase-1 (p < 0.01) in the ALL II group, but there was a significant increase in the expression of NLRP3 in the EV, ALL I, and ALL II groups (p < 0.01) compared with the POF group.

Ultrastructural features of autophagy in the ovary cells of the POF rat

**Figure 11:**
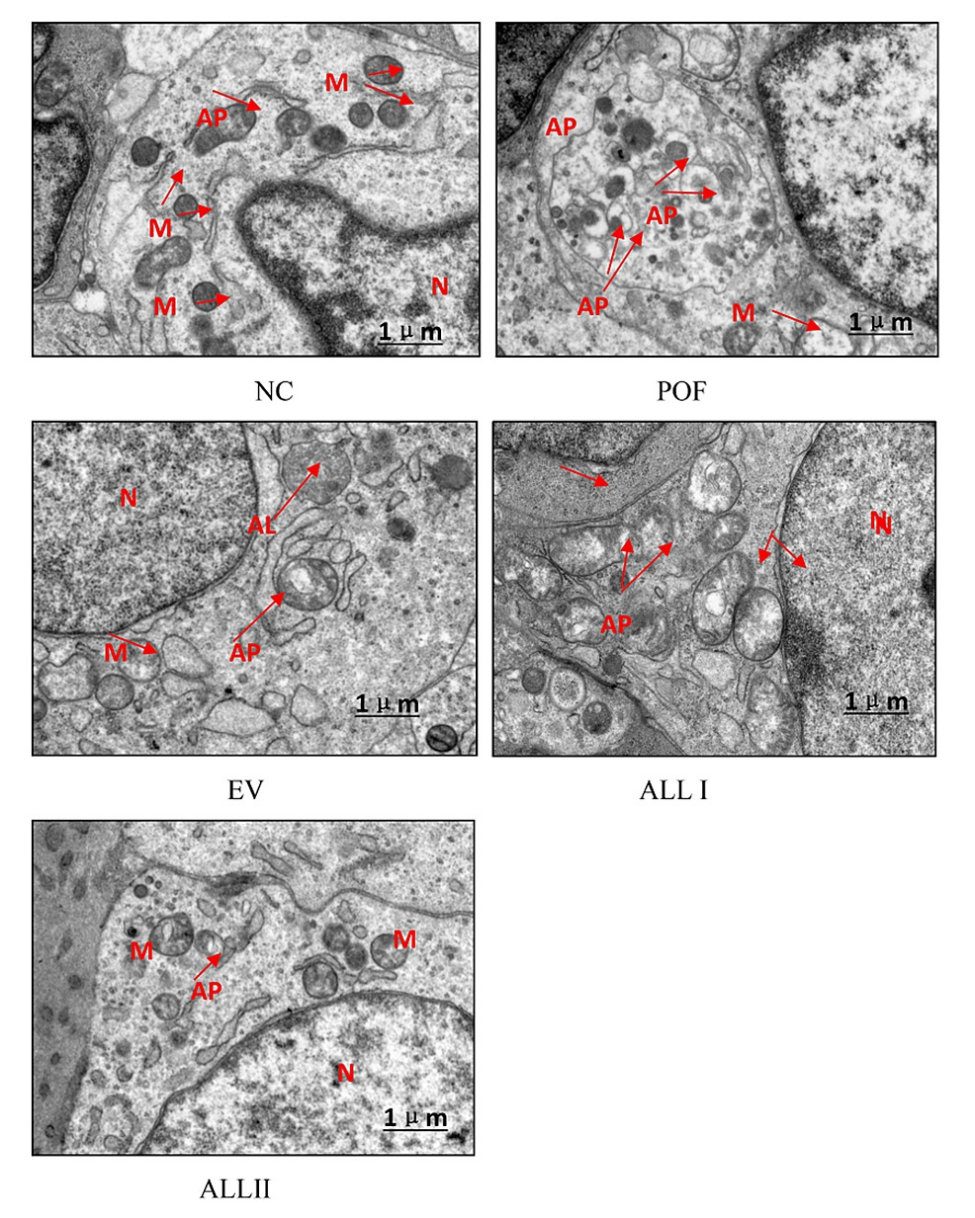
Electron microscopy images of ultrastructural features of autophagy in the ovary cells of the POF rat N: nucleus, AP: autophagosome, AL: autolysosome, M: mitochondria NC: normal control group; POF: premature ovarian failure group; EV: estradiol valerate group; ALL I: the 140 mg/kg dose of allantoin group; ALL II:  the 70 mg/kg dose of allantoin group

Transmission electron microscopy (TEM) was used to determine the presence of autophagosomes (AP) and autolysosomes (AL) in the follicles. TEM showed differences in each group. Healthy follicles of the normal group maintained a close relation with somatic cells; their cytoplasm was homogeneous, the mitochondrial structure was almost normal and the autophagy occurred occasionally. On the contrary, follicles of the POF group displayed abundant autophagic vesicles containing highly degraded cytoplasmic material and mitochondria; the membrane and inner stroma in deformed mitochondria were absent. The EV and ALL can improve the autophagy triggered by the stress response, and the mitochondria were repaired, as shown in Figure 11.

## Discussion

Cyclophosphamide is a chemotherapy drug widely used in the treatment of malignant tumors and autoimmune diseases, but its toxic side effects are strong and can cause permanent damage to the ovary [21]. In the past few decades, the protection of ovarian function has become very important during cancer treatment with the improvement of the quality of life of female cancer patients [22,23]. Follicular atresia is considered one of the main processes for granulosa cell injury leading to premature ovarian failure. The cellular mechanisms of follicular atresia would induce the apoptosis of granulosa cells (GCs), the apoptosis of GCs is induced by a multitude of factors: hormones, reactive oxygen species (ROS), growth factors, cytokines, and Bcl2 family members [24]. Large numbers of studies showed that autophagy maintains the energy balance and function of cells and controls the fate of cells through different crosstalk signals, which may have an important impact on female reproductive function [25]. However, the regulation of the molecular mechanism between apoptosis and autophagy of granulosa cells has not been described fully, and the natural protective agent of granulosa cells on the target of programmed cell death (PCD) has been given a lot of attention [26,27].

Previous studies show that allantoin has estrogen-like effects and modifies oxidative stress in rats with premature ovarian failure. Nonetheless, the mechanism of the allantoin has not been described fully. Therefore, this research focuses on the mechanism of allantoin action in apoptosis, autophagy, and pyroptosis, related to PCD. We detected the apoptosis and autophagy by flow cytometer; similarly, we detected the expression of Bax, Bcl2, and LC3B-II/LC3B-I in order to further research the molecular mechanism. Additionally, this research detected the expression of IL-1β, caspase-1, and NLRP3, which are proteins of the pyroptosis pathway, in order to explore the different types of PCD.

Firstly, allantoin supplementation protects the ovarian reserve from CP-induced ovarian damage, resulting in an estrous cycle, endocrine function, follicle development, and ovary histopathological changes. Allantoin could reduce the anoestrum of the POF rats, increase the level of E2 and decrease the levels of FSH and LH, reduce atretic follicles, and increase mature follicles, primordial follicles, and secondary follicles; the better dose is 140 mg/kg.

Further, we focused on the apoptosis, autophagy, and pyroptosis of granulosa cells. PCD is essential for organism development and homeostasis maintenance, and mechanisms of different PCD have been studied, such as apoptosis, pyroptosis, autophagy, and so on, providing new ideas for the role of different forms of cell death in related diseases and searching for potential drug targets. In our study, the apoptosis rate of the POF group was significantly increased, and the allantoin could reduce cell apoptosis. Mitochondria are organelles that play an essential role in the death of the cell, and are surrounded by a double membrane system, the mitochondrial outer (MOM) and inner membranes (MIM). The decrease in mitochondrial membrane potential (MMP) indicates the early apoptosis of cells [28]. In our study, CP could decrease the MMP of granulosa cells, which indicated that CP induces apoptosis of granulosa cells by damaging mitochondria. The Bcl2 and Bax were detected, and the results show that CP could increase the Bax expression and decrease the Bcl2 expression. Likewise, allantoin could reduce apoptosis and increase autophagy. On the other hand, allantoin could increase the expression of Bcl2 but it has no significant effect on the expression of Bax, which indicates that ALL does not increase MMP by the Bcl2 pathway. Therefore, this study detected the oxidative stress and autophagy of the granulosa cells. The results show that CP could increase ROS and autophagy, and allantoin could protect the granulosa cells by reducing autophagy and the level of ROS.

Pyroptosis is a kind of inflammatory programmed death. The activation of caspase-1 is the key to the classical pyroptosis pathway, which is a defense mechanism against infection [29-31]. In our study, CP could activate the caspase-1 and produce mature IL-1β, and allantoin could improve the inflammatory reaction. However, the expression of NLRP3 has the opposite result, which may be due to excessive autophagy [32]. The results were confirmed by TEM. Allantoin could improve the CP-induced cytotoxicity of granulosa cells by different pathways regulating apoptosis, autophagy, and pyroptosis; the mutual influence and regulation need to be further studied. This study limits the antagonistic experiment of cells and rats, identifying the pathways of the mechanism, and needs to be further studied.

## Conclusions

In this study, we found that the ovarian protective effect of allantoin was associated with the activation of apoptosis, autophagy, and pyroptosis. Allantoin could alleviate cyclophosphamide-induced premature ovarian failure in female rats, decrease anoestrum, increase E2 levels, decrease the levels of FSH and LH, ameliorate apoptosis, and decrease MMP, ROS, and mitophagy in ovarian granulosa cells of POF rats. Further, allantoin down-regulated L-1β, caspase-1, LC3B-II/LC3B-I in ovarian tissue and up-regulated the Bcl2 and NLRP3. Additionally, it would be interesting to conduct more studies on the negative feedback regulation mechanism of NLRP3, which could be related to the immune and stress functions of the organism.
